# Pedicled Instep Flap and Tibial Nerve Reconstruction in a Cynomolgus Monkey* [Macaca fascicularis]*

**DOI:** 10.1155/2016/4386249

**Published:** 2016-10-16

**Authors:** Ruth Weiss, Reto Wettstein, Elisabeth Artemis Kappos, Björn Jacobsen, Daniel Kalbermatten, Alessandra Bergadano

**Affiliations:** ^1^Roche Pharma Research and Early Development, Pharmaceutical Sciences, Comparative Medicine, Roche Innovation Center, Basel, Switzerland; ^2^Department of Plastic, Reconstructive, Aesthetic and Hand Surgery, University Hospital of Basel, Basel, Switzerland; ^3^Roche Pharma Research and Early Development, Pharmaceutical Sciences, Toxicology and Pathology, Roche Innovation Center, Basel, Switzerland

## Abstract

A male cynomolgus monkey experienced extensive soft tissue trauma to the right caudal calf area. Some weeks after complete healing of the original wounds, the monkey developed a chronic pressure sore on plantar surface of the heel of its right foot. A loss of sensitivity in the sole of the foot was hypothesized. The skin defect was closed by a medial sensate pedicled instep flap followed by counter transplantation of a full thickness graft from the interdigital webspace. The integrity of the tibial nerve was revised and reconstructed by means of the turnover flap technique. Both procedures were successful. This is an uncommon case in an exotic veterinary patient as it demonstrates a reconstructive skin flap procedure for the treatment of a chronic, denervated wound in combination with the successful reconstruction of 2.5 cm gap in the tibial nerve.

## 1. Introduction

A male, 6-year-old cynomolgus monkey* (Macaca fascicularis)*, weighting 8.5 kg, suffered extensive soft tissue injuries to the right caudal calf secondary to a bite wound from another monkey. The injuries included the partial transection of the gastrocnemius muscle and the complete transection of the Achilles tendon. The latter remained dysfunctional after complete healing of the original wounds. Several weeks after resolution of the original lesions the monkey developed a chronic wound on the plantar surface of the heel of its right foot. The wound resisted all attempts of conservative treatment following the principles of standard wound care. Although during the course of the disease the animal temporarily demonstrated nonweight bearing lameness, the exposed wound seemed remarkably insensitive to the load of body weight. All these factors combined supported the hypothesis of a loss of sensitivity to the sole of the foot. The plantigrade locomotion in combination with the dysfunctional Achilles tendon and the resulting immobility of the heel further aggravated the pressure on the wound. Based on the clinical finding and the evolution of the wound the following surgical procedures were planned: coverage of the heel wound with a “pedicled instep flap,” exploration, and possible reconstruction of a tibial nerve injury using the “turnover flap technique.”

The animal presented in this case report is part of a group of animals instrumented with telemetric implants allowing measuring hemodynamic parameters in free-moving animals for cardiovascular safety pharmacology studies. Therefore the value of the animal justified the efforts bestowed on its full recovery as well as the potential discomfort associated with the surgery and postoperative recovery phase. All animals were housed in an AAALAC accredited facility and according to the Swiss animal welfare legislation. The NHPs lived in highly hierarchized social groups of 3 to 8 individuals, exclusively indoor in big pens of concrete and metal in one air-conditioned barrier unit. Room temperature was kept constant between 22.5°C and 22.8°C and relative humidity between 50 and 60% (range 40 to 80%), with artificial and natural light. They were fed a laboratory monkey food (70–100 g/day/animal) and daily selection of fresh vegetables. Popcorn and alternatively a mix of cereals, soja, and sunflower seeds were given freely on the ground, once weekly as part of the olfactory enrichment program. Raspberry syrup in water and dry raisins, peanuts, and jelly sweets (Haribos®) were also given weekly during their training sessions as positive reinforcement. Nonchlorinated drinking water (IWB, Basel-Stadt) was available ad libitum.

A structured enrichment program was available with structural elements always present in the pen (ladders, barrels, and sand box) and hanging or free items (kongs, nut cages, puzzle feeders, paint and paper rolls, pet-bottles, and surprise boxes) as auditive (TV) and olfactory (popcorn) elements on a rotational weekly base. The structural elements are used also as visual barriers additional to colored divisions.

Serological testing for tuberculosis, measles, cercopithecine herpes I, and simian viruses (SIV, SRV, and STLV) was performed annually while complete blood counts and clinical chemistries, diabetic markers, and bacteriological and parasitological fecal examination were performed quarterly as part of clinical health checks.

## 2. Case Presentation

### 2.1. Balanced Anesthesia and Analgesia Protocol

After intramuscular sedation (Ketamine/Ketasol-100® and Midazolam/Dormicum®) anesthesia was induced with intravenous administration of alfaxalone (Alfaxan®); after endotracheal intubation anesthesia was maintained with sevoflurane (Sevoflurane®) a popliteal nerve block (lidocaine 2%/Xylocaine 2%) as well as fentanyl (Fentanyl®) CRI ensured perioperative analgesia. Postoperatively carprofen (Rimadyl®) and buprenorphine (Temgesic®) were administered as needed based on a numeric pain score for nonhuman primates (NHPs) developed in house.

### 2.2. Surgical Procedure “Pedicled Instep Flap”

**Figure 1:**
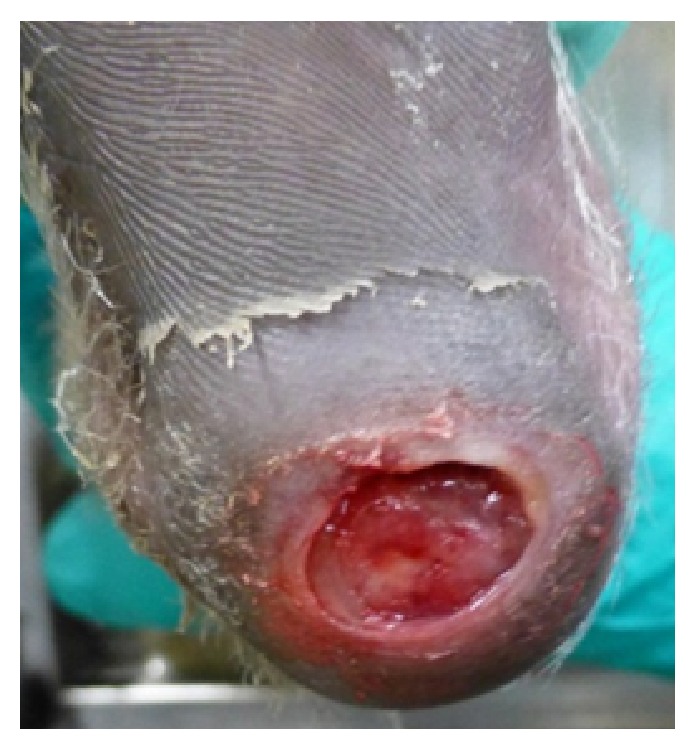
2.5 × 3.5 cm lesion situated in the middle of the weight bearing part of the heel of the foot.

The lesion was situated in the middle of the weight bearing part of the heel of the foot, about 2.5 × 3.5 cm in size (Figure 1).

**Figure 2:**
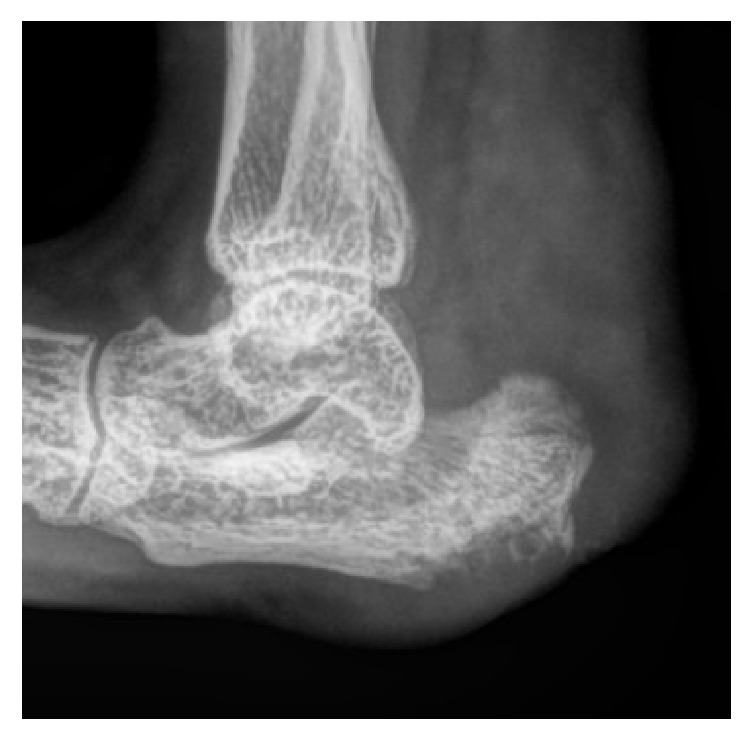
Radiographs of the animal's foot taken 26 days before the surgical intervention.

Thereby it compromised nearly 90% of the weight bearing part of the animal's heel. The calcaneal bone was covered merely by nonviable granulation tissue. Radiographs revealed inflammation of the adjoining structures, that is, the soft tissues of the ankle joint as well as probable osteomyelitis in the calcaneal bone (Figure 2).

**Figure 3:**
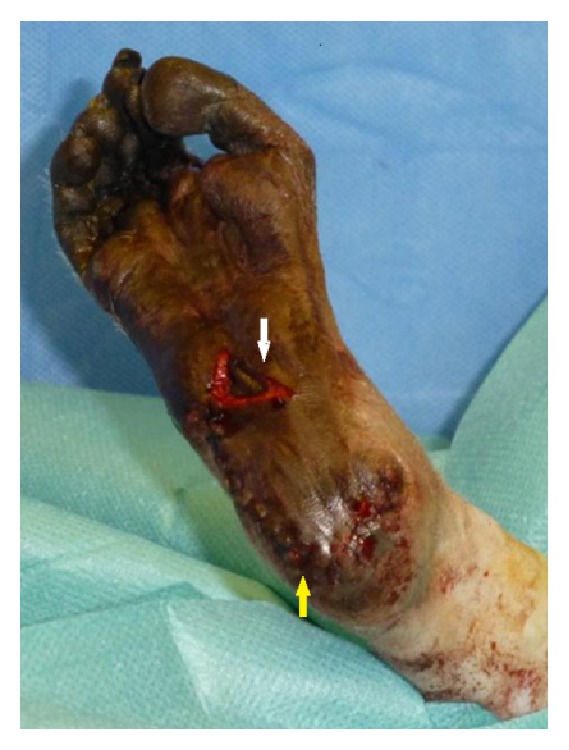
Pedicled instep flap (yellow arrow), donor site covered by counter transplantation of a full thickness graft from the interdigital webspace in between the digiti pedis I and digiti pedis II of the ipsilateral foot (white arrow).

The animal was placed in a prone position. The surgical area was prepared in an aseptic manner. The heel flap operation was performed under tourniquet control. After debridement and reconditioning of the wound, the macroscopically affected part of the calcaneal bone was removed by means of bone rongeur. In the process superficial as well as deep bone biopsies were taken. Histopathologically, the removed part of the calcaneus showed a superficial massive fibrosis mixed with small vessels indicative for mature granulation tissue as well as a sparse infiltration of mononuclear inflammatory cells. A slow-release, self-dissolving gentamycin sponge (Septocoll®E 40, Biomet) was inserted into the resulting bone defect. After localization of the medial plantar artery via Doppler, an adequately sized flap in relation to the animal's heel surface was dissected around the instep area, to ensure complete coverage of the heel lesion without tension. The neurovascular pedicle was preserved containing superficial branches of the median plantar artery as well as cutaneous branches of the median plantar nerve. The flap was positioned over the defect and attached to the surrounding tissue via continuous subcutaneous followed by continuous intracutaneous sutures (both Vicryl 4.0) and a cutaneous suture (Ethilon 4.0). The donor site was covered by counter transplantation of a full thickness graft from the interdigital webspace in between the digiti pedis I and digiti pedis II of the ipsilateral foot (Figure 3). The graft was fixated via simple interrupted sutures (PDS 5.0).

After surgery the affected foot was protected with a half-cast for approx. 5 weeks. Until sensitivity had recovered the foot was continuously covered with a light protection bandage.

### 2.3. Surgical Procedure Tibial Nerve Reconstruction

**Figure 4:**
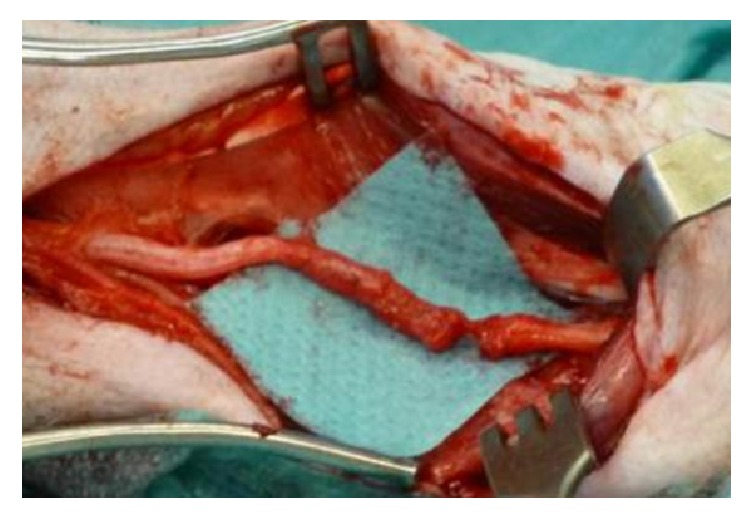
Complete transection of the tibial nerve in the midcalf area (intraoperative image).

Based on the location of the original wounds an injury to the tibial nerve was possible in several locations. The tibial nerve was explored and eventually found severed in the midcalf area, allegedly distal to the motor branches of the nerve (Figure 4).

According to Sunderland's classification the injury corresponded to a grade V injury [1]. Histopathology confirmed the expected degeneration of the distal nerve stump consisting of mature fibrous tissue mixed with inflammatory cells. On the proximal stump, traumatic neuroma formation was suspected from macroscopic appearance. Histopathologically, loose nerval tissue with the presence of vital axons, Schwann cells, and myelin sheaths embedded in mature fibrous tissue were detected. Reconditioning of the stumps, that is, removal of scar tissue and trimming to healthy fascicular structures, resulted in a gap of approximately 25 mm. Under 2.5 magnification the tibial nerve was dissected. After careful preparation of the fascicles and splitting of the distal nerve stump a turnover flap was formed. The nerve flap was turned over to reach the proximal end of the nerve. This way the integrity of the nerve was reconstructed by means of the “fascicular turnover flap method” developed by Koshima et al. [2]. This technique allowed for tension-free bridging of the defect. Coaptation of the nerve flap was performed using epineural sutures (Nylon 8.0). The wound was closed via continuous subcutaneous followed by continuous intracutaneous sutures (both Vicryl 4.0) and a cutaneous suture (Ethilon 4.0).

### 2.4. Follow-Up Phase

Postoperatively the monkey received a veterinary formulation of amoxicillin/clavulanic acid (Synulox®) in total for 12 weeks based on bacteriology bone culture results. The postoperative phase of both wounds was uneventful apart from a slight delay in wound healing on the most lateral portion of the heel flap.

**Figure 5:**
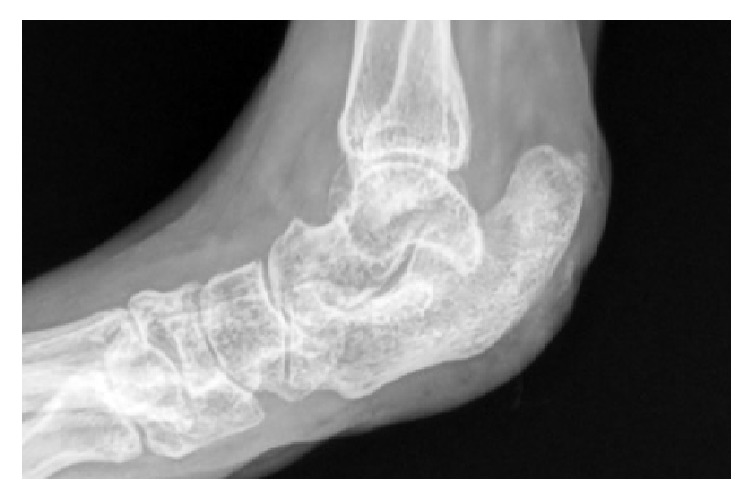
Control radiographs (33 days after surgery) illustrating the normalization of the calcaneal bone as well as the soft tissue structures around the ankle joint.

33 days after surgery control radiographs illustrated the normalization of the calcaneal bone structure as well as the soft tissue around the ankle joint (Figure 5).

84 days after surgery return of sensitivity to the plantar surface—in particular the flap area—could be objectively established by means of selective thermal stimulation of A*δ* and C fibers. The flap area and the contralateral uninjured heel (acting as own control) were stimulated with a pen-type thermal stimulator. The hand-held pen (contact area of approx. 0.5 cm^2^) was applied to the skin of the heel and the temperature gradually increased with a rate of 6.5°C/s (cut-off 55°C) until a withdrawal reflex was elicited consisting of flexion of the ankle and knee joints. Reproducible thresholds could be elicited upon stimulation of the transplanted flap area compared to the contralateral healthy heel area.

During the weeks following the bandage removal, function and load on the recovering foot gradually improved. At long-term follow-up, more than one year after the intervention the flap was visually indistinguishable from the surrounding skin and its durability and function seemed equal to that of normal plantar skin. Functionally, only a decrease in range of motion of the toes was persisting and when running the animal intermittently favored the formerly injured foot. However, most of the residual gait abnormality was due to the dysfunctional Achilles tendon. About 1 year after full recovery the animal had to be euthanized due to reasons unrelated to the injuries presented in this case report. In the autopsy the reconstructed nerve section was found to be completely healed.

## 3. Discussion

In the case presented here, impairment in wound healing was apparent, characterized by the lack of wound contraction and a sluggish formation of granulation tissue. This is in striking contrast to the usually excellent healing potential of traumatic injuries in NHPs. The shortcomings in wound healing were tentatively related to the denervated state of the injured skin in addition to the inauspicious conditions, that is, exposure to wear and body excretions. The absence of sensitivity to the sole of foot was confirmed later when the tibial nerve (N) was found severed. Trophic ulceration of digital pads has been described in veterinary medicine [3–5]. It is well established that in clinical practice wound healing is delayed in denervated skin areas even without additional hindering factors such as pressure and/or impaired vascularization (i.e., diabetes). There are striking features in biochemical wound characteristics that can be linked to denervation such as depleted levels of nerve growth factor,* substance P* and* calcitonin gene-related peptide* [6]. Fujiwara et al. demonstrated in a novel* in vitro* model the role of neuronal processes in the differentiation of fibroblasts to myofibroblasts and in consequence secretion of collagen fibers essential for wound contraction [7]. Animal models support these observations. In denervated wounds in rats Richards et al. demonstrated reduced neuropeptide concentration, reduced monocyte, and macrophage as well as T lymphocyte counts and in consequence reduced monocyte and macrophage chemotaxis. Delay in wound healing was illustrated by recording of delayed wound contraction [8].

The anatomic structure of the plantar surface of the primate foot as the main weight bearing body surface represents a highly specialized architecture to be able to buffer the pressure and shear forces. The glabrous epidermis and dermis are much thicker than in any other part of the body. Vertical fibrous septa extend from the dermis to the underlying fascia subdividing subcutaneous fat into discrete compartments. Reconstructions of defects to the plantar skin are technically challenging, as skin transplanted from other regions of the body is unable to withstand the strain of weight bearing. The instep area however represents the ideal donor site; while it provides the necessary anatomical characteristics, it is not needed for weight bearing [9, 10]. The medial plantar artery and parts of the N. plantaris medialis are included in the neurovascular pedicle of the flap. Neurovascular free and island flaps have been applied successfully in veterinary medicine in order to replace injured canine foot pads [3, 5, 11]. In human patients Wan et al. could demonstrate at 6 months to 1 year after sensate medial plantar flap transposition that the sensation of the recipient area retained the quality of the donor site, thereby surpassing the sensation of the contralateral normal heel [12]. Differences can be found in the quality of sensation such as temperature, pressure, and two-point discrimination [13]. Then again in a long-term comparison noninnervated free flaps (spontaneous reinnervation) have not proven to be superior to reinnervated free flaps (sensory nerve preservation or coaptation) [14]. However, in our animal patient regain of sensation as soon as possible was mandatory to prevent traumatic reinjury and therefore enhance flap durability, since the lack of sensation was likely the cause of the pathology in the first place. It was believed that, by perpetuating the continuity of sensory nerve structure into the flap, nerve regeneration would be facilitated, even though the underlying impediment was the midcalf tibial nerve injury.

The prognosis for bridging its 25 mm defect without surgical intervention would have been poor. In order to provide a scaffold for smooth and unbranched axoplasmic migration into the Schwann cell tube of the distal stump, the continuity of the severed N. tibialis had to be reconstructed. The surgical reconstruction was performed approx. 15 weeks after the original injury had occurred. After a delay of more than 8 weeks axoplasmic recannulation may be hindered due to stromal fibrosis and narrowing of Schwann cells in the distal stump. The rather novel fascicular turnover flap technique applied in this case was first introduced by Koshima and coauthors in 2010 [2]. It allows for bridging of nerve defects over 20 mm in length using the injured nerve as its own donor nerve. As a special asset this technique results in a vascularized nerve graft. A free nerve graft has to rely on the vascularization of the recipient bed until spontaneous revascularization occurs. In veterinary medicine autologous free nerve grafting has been applied with satisfactory results [15]. However, the vascularization of nerve grafts becomes principal, where thick grafts are involved, in large defects or in cases such as ours where the graft meets up with a scarred recipient bed; the longer the gap the higher the risk of atrophy as well as fibrous ingrowth into the distal anastomosis area [16]. Vascularization of nerve grafts has been linked to suppression of fibrosis and leads to a swifter recovery and a better end result concerning sensorimotor nerve function [17, 18]. The expected speed of nerval regeneration is about 1–3 mm/day.

When the pressure sore first developed we unsuccessfully tried to test for sensibility in the affected region by means of skin pinching and/or needle picking. While being successfully applied in small animal patients such as dogs and cats, the sensitivity testing based on mechanical pinching and needle picking in this case yielded only inconclusive results. Therefore up to the surgery and the visual revision of the tibial nerve the loss of sensitivity remained a hypothesis. About 3 months after surgery, return in nociceptive sensitivity in the injured heel could be assessed using selective thermal stimulation of A*δ* and C fibers which elicited a reproducible withdrawal reflex. Heat pain activates A*δ* or C nociceptors depending on the intensity of the stimulus and whether the skin is heated at a rapid or a slow rate, respectively. The use of thermal stimulation is a model of phasic pain and allows for exploring the peripheral nociceptive pathways [19–21]. We can speculate that also tactile sensitivity carried by the A*β* fibers was restored anatomically and physiologically.

In addition to the systemic medication a gentamycin sponge (Septocoll E 40) was fitted into the mold in the calcaneal bone that had resulted from bone debriding. Antibiotic-impregnated beads had already been used successfully in a case of calcaneal osteomyelitis resistant to conventional medical therapy in a Rhesus Macaque [22]. However, neither histopathology nor microbiologic culture from the specimens taken at the moment of surgery was able to confirm active infection as a cause for the delay in wound healing.

## 4. Conclusion

This is a unique report in veterinary medicine describing the successful surgical repair of a chronic plantar defect by the “pedicled instep flap” technique and the reconstruction of the tibial nerve. It demonstrates the consequence of denervation on the development and evolution of a skin defect in the tibial nerve's autonomous zone, the sole of the foot. Thereby it brings to mind the crucial part of innervation in the process of wound healing.
